# MicroRNA profiling in human diploid fibroblasts uncovers miR-519 role in replicative senescence

**DOI:** 10.18632/aging.100159

**Published:** 2010-06-19

**Authors:** Bernard S. Marasa, Subramanya Srikantan, Jennifer L. Martindale, Mihee M. Kim, Eun Kyung Lee, Myriam Gorospe, Kotb Abdelmohsen

**Affiliations:** ^1^ Laboratory of Cellular and Molecular Biology, NIA-IRP, NIH, Baltimore, MD 21224, USA; ^2^ Department of Biology, The Catholic University of America, Washington, DC 20064, USA

**Keywords:** HuR, miR-519, tumor suppression

## Abstract

MicroRNAs (miRNAs) are short non-coding RNAs that regulate diverse biological processes by controlling the pattern of expressed proteins.  In mammalian cells, miRNAs partially complement their target sequences leading to mRNA degradation and/or decreased mRNA translation.  Here, we have analyzed transcriptome-wide changes in miRNAs in senescent relative to early-passage WI-38 human diploid fibroblasts (HDFs).  Among the miRNAs downregulated with senescence were members of the let-7 family, while upregulated miRNAs included miR-1204, miR-663 and miR-519.  miR-519 was recently found to reduce tumor growth at least in part by lowering the abundance of the RNA-binding protein HuR.  Overexpression of miR-519a in either WI-38 or human cervical carcinoma HeLa cells triggered senescence, as measured by monitoring β-galactosidase activity and other senescence markers.  These data suggest that miR-519 can suppress tumor growth by triggering senescence and that miR-519 elicits these actions by repressing HuR expression.

## Introduction

MicroRNAs are short (~ 22-nt) RNA molecules that modulate changes in gene expression [1,2].  They are generated from precursor transcripts (primary microRNAs) which are exported to the cytoplasm and are cleaved by Dicer; mature miRNAs then assemble into ribonucleoprotein silencing complexes (RISC) that are recruited to specific mRNAs [3].  MicroRNAs function primarily as repressors of mRNA stability and translation [4].  Through their influence on the patterns of expressed genes, microRNAs have been implicated in numerous physiologic processes, such as develop-ment of the muscular, immune, neuronal, epithelial and other systems, and in pathologies including neuro-degeneration and cancer [5-8].  The latter studies have revealed a number of miRNAs that can function as tumor suppressors (TS-miRNAs) or tumor promoters (oncomiRs) [9].

Cellular senescence is achieved when cells reach the end of their replicative lifespan [10,11]. It is believed to represent a tumor-suppressive mechanism and a contributing factor in aging [12,13].  MicroRNAs have been implicated in replicative senescence, since loss of miRNA biogenesis through Dicer ablation causes senescence in primary cells [14].  Several specific miRNAs were reported to be differentially expressed in senescent cells compared to young, proliferating cells.  For example, miRNA-146a and miR-146b are up-regulated in senescent cells and modulate inflammatory responses by suppressing secretion of IL-6 and IL-8 and by downregulating IRAK1 [15].

Recently, four microRNAs (miR-15b, miR-24, miR-25, and miR-141) that jointly lower expression of the kinase MKK4 were found to decline during replicative senescence and to contribute to the senescence process [16].  miR-24 was also found to regulate translation of the cyclin-dependent kinase inhibitor p16, thereby allowing increased p16 expression in senescent cells [17].

Several miRNAs differentially expressed with aging have also been identified.  For example, miR-17, miR-19b, miR-20a, and miR-106a were less abundant in cells from older humans [18]. Reduced expression of miR-103, miR-107, miR-128, miR-130a, miR-155, miR-24, miR-221, miR-496, and miR-1538 in older individuals was also recently reported [19].  Age-regulated changes in the expression of microRNAs were also found in mouse liver and brain [20,21].  MicroRNA changes in Ames dwarf mouse liver led to the identification of microRNAs that might delay aging [22].  Studies in *Caenorhabditis elegans* revealed that the microRNA *lin-4 *represses *lin-14* transcripts and lin-14 protein to extend lifespan by reducing DAF-16; miRNA profiling in *C elegans* provided evidence that microRNAs may potently influence the biology of aging [23-25].

Many studies have focused on the role of microRNAs in tumorigenesis and age-related diseases.  Here, we have studied changes in expressed microRNAs during replicative senescence of WI-38 human diploid fibroblasts (HDFs).  We identified subsets of microRNAs that were differentially expressed in young compared with senescent WI-38 cells.  miR-519, a microRNA that suppresses tumorigenesis and lowers expression of RNA-binding protein HuR, was upregulated in senescent cells.  Overexpression of miR-519 induced senescence in WI-38 and HeLa cells.  Our data support the hypothesis that senescence-associated changes in microRNA expression patterns can affect the susceptibility to age-related diseases such as cancer.

**Figure 1.**
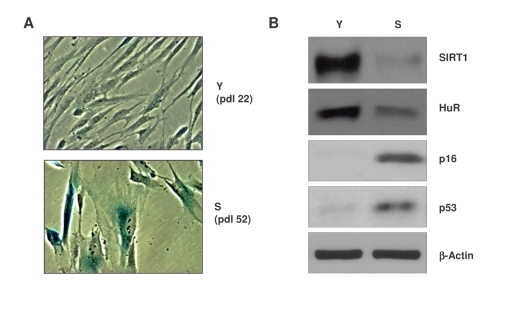
Characterization of early-passage and senescent WI-38 cells. **(A)** Micrographs illustrating β-galactosidase activity in young (Y) early-passage (pdl 22) and senescent (S), late-passage (pdl 52) WI-38 cells.  (**B**) Western blot analysis of the proteins indicated in whole-cell lysates prepared from Y and S WI-38 populations;  β-actin served as a loading control.

## Results

### Global changes in microRNAs between early-passage and senescent WI-38 human diploid fibroblasts

Compared with early-passage, ‘young' proliferating [Y, at population doubling (pdl) 22] WI-38 cells, the senescent (S, pdl 52) WI-38 cells displayed a flattened morphology and senescence-associated (SA) β-galactosidase (SA-β-gal) activity, a widely used senescence marker [26,27] (Figure 1A).  Western blot analysis also revealed that senescent cells expressed lower levels of SIRT1 and HuR, whereas p16 and p53 were upregulated (Figure 1B), in keeping with reported literature [28-30].

**Figure 2.**
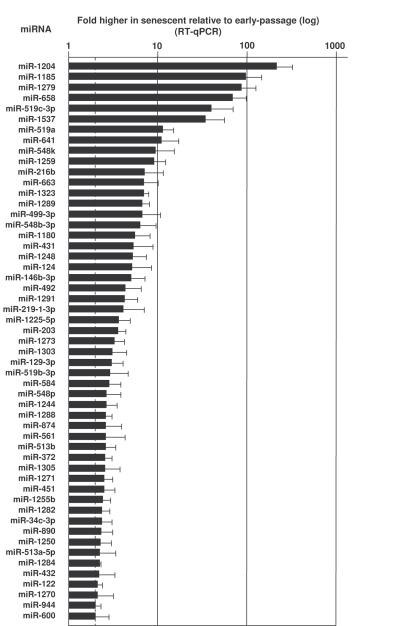
MicroRNAs upregulated in senescent cells. RNA extracted from Y (pdl 22-25) and S (pdl 50-55) WI-38 cells was used to measure the levels of the microRNAs listed, using RT-qPCR (Materials and Methods).  MicroRNA abundance was normalized to U1 snRNA levels.  Data are the means and S.D. from three independent experiments.

**Figure 3.**
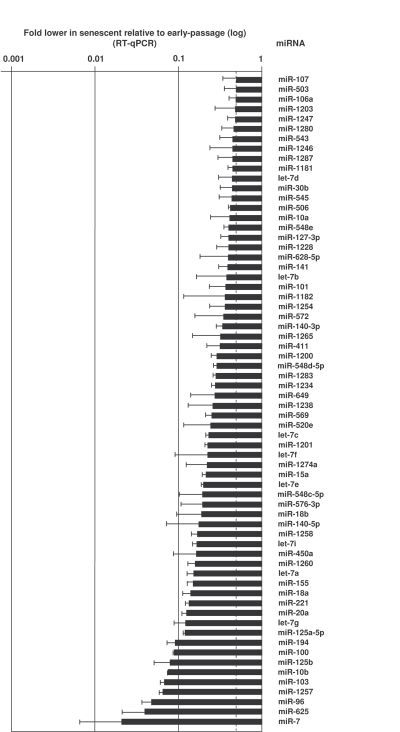
MicroRNAs downregulated in senescent cells. RNA was extracted and analyzed as explained in the legend of Figure 2. Data show the means and S.D. from three independent experiments.

To test how the pattern of expressed microRNAs is affected by replicative senescence, we studied transcriptome-wide changes in microRNAs using miRNome arrays (not shown); we then validated individual microRNAs by reverse transcription (RT) followed by real-time, quantitative (q)PCR amplification (see Materials and Methods).  Depicted in Figures 2 and 3 and in Supplementary Table 1 are all of the microRNAs validated using sequence-specific qPCR primers.  As shown in Figure 2, several microRNAs were markedly more abundant in senescent cells (e.g., miR-1204, miR-663, miR-548b-3p and miR-431).  Other microRNAs were expressed at lower levels in senescent cells [e.g., miR-24, miR-141, and miR-10a (Figure 3, Supplementary Table 1)].  MicroRNAs changing less than twofold with senescence are listed in the Supplementary Table 1.

**Figure 4.**
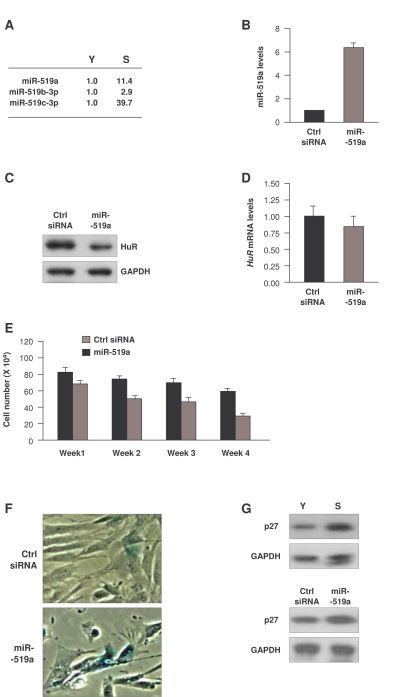
Influence of miR-519 on WI-38 senescence. **(A)** Fold differences in miR-519 expression in S relative to Y cells, calculated as explained in the legend of Figure 2. ** (B)** Forty-eight h after transfection of either control (Ctrl) siRNA or miR-519a, the levels of miR-519a were measured by RT-qPCR.  **(C,D)** In cells transfected as explained in panel (B), the levels of HuR protein and loading control GAPDH were assessed by Western blot analysis (C), and the levels of *HuR* mRNA and normalization control 18S rRNA were measured by RT-qPCR (D).  **(E)** WI-38 cell numbers in cells transfected as in (B) were counted every 7 days.  **(F)** SA-β-galactosidase activity in WI-38 cells by week 4 after sequential transfection (every 7 days) of either Ctrl siRNA or miR-519a.  **(G)** Western blot analysis of p27 and loading control GAPDH in Y and S WI-38 cells (*top*) or in WI-38 cells by week 4 after transfection as explained in (E) (*bottom*).  The data in B,D,E represent the means and S.D. from three independent experiments.

### miR-519-induced senescence in HDFs

We were particularly interested in the miR-519 family.  miR-519 was recently found to inhibit translation of the RNA-binding protein HuR through its interaction with the HuR coding region [31].  In a separate study, miR-519 suppressed the growth of tumor xenografts in an HuR-dependent manner [32].  Given that HuR promotes cell proliferation and decreases senescence [33,34], we hypothesized that the elevated miR-519 in senescent cells (Figure 4A) might lower HuR expression in WI-38 HDFs, and hence promote senescence.  To test this possibility, we overexpressed miR-519a in young-HDFs (Figure 4B);  western blot analysis confirmed that miR-519a  overexpression repressed HuR  (Figure 4C).  In keeping with earlier results [31], miR-519a did not influence the levels of *HuR* mRNA (Figure 4D), in agreement with the view that miR-519a inhibited *HuR* mRNA translation without affecting *HuR* mRNA stability.  Moreover, sustained miR-519a overexpression for 4 weeks caused a marked reduction in cell number as compared to control transfection groups (Figure 4E).  miR-519a-overexpressing cells also showed increased SA-β-gal activity (Figure 4F) and elevated expression of the senescence marker p27 [35,36] (Figure 4G, *bottom*). Together, these data indicate that miR-519a induced cellular senescence and inhibited cell proliferation, resulting in accelerated senescence.  They further suggest that miR-519a-induced senescence may be mediated in part by repression of HuR.

**Figure 5.**
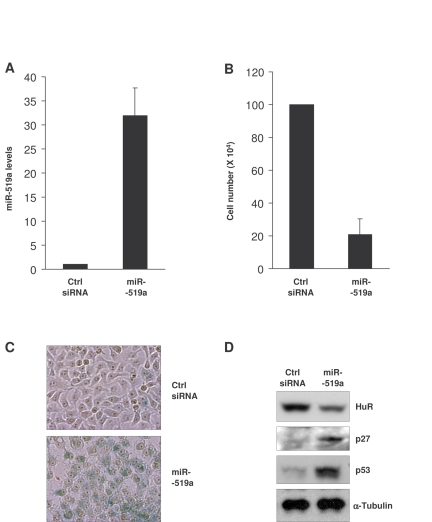
Influence of miR-519 on the senescent phenotype of HeLa cells. **(A)** Forty-eight h after transfection of HeLa cells with either control (Ctrl) siRNA or miR-519a, miR-519a levels were measured by RT-qPCR.  **(B)** Number of HeLa cells remaining by 72 h after transfection of Ctrl siRNA or miR-519a as explained in (**A**).  **(C)** β-galactosidase activity in HeLa cells 5 days after transfection with either Ctrl siRNA or miR-519a.  **(D)** Seventy-two hours after transfection as indicated in (A), the levels of the proteins shown were assessed by Western blot analysis.  The data in A,B represent the means and S.D. from three independent experiments.

### miR-519-induced senescence in HeLa cells

As indicated above, miR-519 was found to suppress tumor growth [32].  Since cellular senescence is considered to be an anti-tumorigenic process, we examined the effect of miR-519 on the senescent phenotype of cancer cells.  Upon miR-519a overexpression (Figure 5A), HeLa cell numbers declined  significantly (Figure 5B).  Five days after transfection of miR-519a, cells showed a strong increase in SA-β-gal activity compared to the control transfection group (Figure 5C);  in addition, miR-519-induced senescence in HeLa cells was accompanied by increased levels of the senescence markers p53 and p27 (Figure 5D).  Together, these data indicate that miR-519 reduced HeLa cell proliferation and promoted HeLa cell senescence. Accordingly, we postulate that one of the mechanisms by which miR-519 suppress tumor growth is by inducing senescence, and further propose that miR-519 triggers senescence -at least in part- by reducing HuR levels.

## Discussion

Cells become senescent as a result of factors such as the accumulation of reactive oxygen species, DNA damage, erosion of telomeres, and oncogenic activation.  Collectively, these triggers cause cells to undergo morphological changes, to become unable to replicate DNA and to display altered gene expression patterns [10-13].  Here, we investigated microRNA levels in WI-38 human diploid fibroblasts by comparing microRNA patterns in senescent relative to young, proliferating cells.  Among the microRNAs showing increasing abundance with senescence, miR-519 was of particular interest because it was shown to inhibit translation of HuR and to diminish tumor growth [31,32]. Through its influence on the expression of many genes, HuR plays a key role in cell proliferation, tumorigenesis, and senescence [37,38].  We found that overexpression of miR-519a decreased HuR levels, lowered cell proliferation, and promoted replicative senescence in both WI-38 and HeLa cells.

### microRNAs and senescence

We previously used miRNA microarrays to identify changes in a limited number of microRNAs in senescent cells [16].  Here, we have expanded this analysis and have verified many individual microRNAs whose abundance changes with replicative senescence.  Many of them target key proteins implicated in senescence and cancer.  For example, miR-146b is upregulated in senescent cells (Figure 2), in keeping with earlier findings that miR-146a and miR-146b increased with senescence and repressed the senescence-associated inflammatory mediators IL-6 and IL-8 [15].  miR-34a regulates SIRT1 expression and induced senescence of cancer cells [39-41]; here, we observed higher miR-34c (not miR-34a) in senescent cells, likely a reflection of the variability and complexity of the senescence process.  Several let-7 members were also upregulated in senescent cells (Figure 2 and Supplementary Table 1);  this observation supports the view that the ability of let-7 microRNAs can suppress tumor growth [reviewed in 42], which could contribute to the senescence process.  Similarly, upregulation of miR-20 in senescence cells correlates with the ability of miR-20 to inhibit proliferation of K562 human erythromyeloblastoid leukemia cells [43].  In conjunction with the finding that miR-519 reduced tumorigenesis in a xenograft model [32], we propose that the coordinated action of senescence-upregulated microRNAs can suppress tumor growth by reducing the levels of oncogenes or tumor promoters.

Conversely, many microRNAs were downregulated in senescent cells (Figure 3).  Among the myriad of senescence-associated proteins that they might regulate, these microRNAs likely repress several tumor suppressors.  In this regard, as miR-21 has been shown to lower expression of the tumor suppressor PTEN [44], the downregulated of miR-21 in senescent cells (Figure 3) could allow increased PTEN expression, in turn reducing tumor cell proliferation, migration, and invasion [45].

### miR-519a-induced senescence by lowering HuR

We previously reported that miR-519 represses the production of HuR, an RNA-binding protein which is highly abundant in cancer cells and is low in untransformed cells [11,38].  HuR overexpression delays the senescent phenotype while the loss of HuR enhances it [11].  Moreover, while HuR levels are high in tumors and low in normal tissues, miR-519 levels are high in normal tissues and low in cancer tissues [32].  Since HuR potently enhances the expression of cancer-promoting proteins, and reducing HuR levels promotes HDF senescence [11,38], we propose that miR-519 represses tumor growth at least in part, by lowering HuR and thereby promoting senescence (Figs. 4 and 5). Additionally, miR-519 could further repress tumor growth by lowering the expression of other genes, such as ABCG2 or HIF-1α [46,47].

In summary, we have identified collections of microRNAs displaying altered abundance with replicative senescence.  As shown here for miR-519, we postulate that these changes help to meet the needs of senescent cells in eliciting tumor suppression and growth arrest.  Future studies will help to recognize more fully the proteins and processes modulated by senescence-regulated microRNAs.

## Materials and methods

Cell culture, transfections, and β -galactosidase staining. Early-passage, proliferating (‘young', ~20 to 30 pdl) and late-passage, senescent (~50 to 55 pdl) WI-38 human diploid *fibroblasts* (HDFs; Coriell Cell Repositories) were cultured in Dulbecco's modified Eagle's medium (DMEM, Invitrogen) supplemented with 10% fetal bovine serum and 0.1 mM nonessential amino acids (Invitrogen).  HeLa cells were cultured in DMEM supplemented with 10% FBS and antibiotics.  miR-519a (Ambion) or control siRNA (AATTCTCCGAACGTGTCACGT, Qiagen) were transfected at a final concentration of 100 nM using Lipofectamine 2000 (Invitrogen).  Where indicated, transfections were performed every 7 days for 4 weeks.  WI-38 HDFs and HeLa cells were stained with a senescence-associated β-galactosidase (Cell Signaling Technology) detection kit, according to the manufacturer's protocol.

RNA isolation and miRNA profiling. Total cellular RNA was isolated using Trizol (Invitrogen).  Isolated RNA was used to measure miRNA levels in young and senescent cells with a 7900HT real-time PCR instrument (Applied Biosystems).  All microRNAs were measured and validated using miRNA-specific forward primers (Supplementary Table 2) and a universal reverse primer (System Biosciences, SBI), according to the manufacturer's protocol.  The levels of U1 snRNA, used for normalization, were determined using the specific forward primer CGACTGCATAATTTGTGGTAGTGG.

Protein analysis. Whole-cell lysates were prepared with RIPA buffer [10 mM Tris-HCl (pH 7.4), 150 mM NaCl, 1% NP-40, 1 mM EDTA, 0.1% SDS, and 1 mM dithiothreitol].  Proteins were resolved by SDS-polyacrylamide gel electrophoresis and transferred to polyvinylidene difluoride membranes (Invitrogen).  After incubation with primary antibodies recognizing SIRT1, HuR, p16, p53, p27, GAPDH (all from Santa Cruz Biotechnology) or β-actin (Abcam), blots were incubated with the appropriate secondary antibodies and the signals were detected by ECL Plus (GE Healthcare).

## Supplementary data

Supplementary Table 1 MicroRNAs showing less than twofold differences in abundance in senescent relative to early-passage cells.

Supplementary Table 2 MicroRNAs showing less than twofold differences in abundance in senescent relative to early-passage cells.
