# Supplementary material for: MicroRNA profiling in human diploid fibroblasts uncovers miR-519 role in replicative senescence
Source: Aging (Albany NY). 2010 Jun 19;2(6):333–43. doi: 10.18632/aging.100159 (PMC2919253; doi:10.18632/aging.100159)
Supplement: Supplementary Table 1 [file aging-02-333-s001.pdf]

miRNA	FC	SD	miRNA	FC	SD	miRNA	FC	SD
miR-605	1.98	0.61	miR-504	1.25	0.35	miR-129-5p	0.98	0.06
miR-1231	1.97	0.10	miR-508-3p	1.24	0.40	miR-518c	0.95	0.40
miR-1256	1.95	0.55	miR-1262	1.24	0.30	miR-640	0.93	0.20
miR-128	1.79	0.26	miR-509-3-5p	1.24	0.32	miR-1243	0.91	0.31
miR-562	1.79	1.11	miR-509-5p	1.23	0.40	miR-653	0.88	0.43
miR-548l	1.79	0.76	miR-511	1.23	0.30	miR-1263	0.83	0.48
miR-520b	1.75	0.59	miR-512-5p	1.23	0.50	miR-618	0.82	0.60
miR-373	1.72	0.75	miR-515-3p	1.22	0.31	miR-106b	0.82	0.60
miR-483-5p	1.71	1.01	miR-513c	1.22	0.41	miR-135a	0.81	0.51
miR-550	1.71	1.07	miR-516b	1.22	0.22	miR-548d-3p	0.81	0.13
miR-25	1.70	0.75	miR-516a-3p	1.22	0.21	miR-520a-3p	0.80	0.44
miR-518e	1.68	0.53	miR-517b	1.22	0.51	miR-651	0.79	0.35
miR-1224-3p	1.66	0.52	miR-518a-3p	1.21	0.24	miR-133b	0.78	0.17
miR-876-5p	1.66	0.52	miR-518b	1.21	0.36	miR-1202	0.78	0.07
miR-507	1.64	1.04	miR-518d-3p	1.21	0.51	miR-1197	0.78	0.28
miR-1266	1.64	0.57	miR-520a-5p	1.17	0.40	miR-548i	0.77	0.15
miR-577	1.64	0.13	miR-494	1.16	0.20	miR-548c-3p	0.77	0.19
miR-1304	1.62	0.76	miR-520g	1.15	0.31	miR-520d-3p	0.75	0.14
miR-1253	1.59	0.41	miR-523	1.15	0.30	miR-34a	0.75	0.38
miR-760	1.58	1.14	miR-521	1.15	0.29	miR-664	0.74	0.17
miR-519e	1.58	1.02	miR-524-5p	1.15	0.50	miR-1208	0.73	0.13
miR-206	1.57	1.07	miR-525-5p	1.15	0.46	miR-9	0.73	0.13
miR-127-5p	1.54	0.75	miR-526b	1.15	0.44	miR-1268	0.72	0.15
miR-1227	1.50	0.11	miR-532-3p	1.15	0.43	miR-217	0.72	0.02
miR-370	1.50	0.45	miR-539	1.14	0.33	miR-1237	0.70	0.04
miR-371-5p	1.49	0.51	miR-542-3p	1.14	0.63	miR-758	0.70	0.15
miR-1207-3p	1.48	0.11	miR-548b-5p	1.12	0.23	miR-548h	0.70	0.15
miR-510	1.48	0.32	miR-633	1.11	0.40	miR-495	0.69	0.13
miR-888	1.47	1.10	miR-208a	1.10	0.07	miR-1229	0.67	0.10
miR-376a	1.46	0.71	miR-548g	1.10	0.20	miR-1236	0.66	0.13
miR-376c	1.46	0.52	miR-1276	1.10	0.14	miR-320c	0.65	0.10
miR-378	1.46	0.76	miR-1269	1.10	0.52	miR-1179	0.65	0.07
miR-1272	1.46	0.08	miR-508-5p	1.10	0.44	miR-24	0.64	0.21
miR-380	1.46	0.39	miR-549	1.10	0.32	miR-1281	0.63	0.13
miR-553	1.46	0.18	miR-1826	1.09	0.68	miR-548a-5p	0.62	0.13
miR-382	1.45	0.60	miR-1266	1.09	0.20	miR-212	0.61	0.13
miR-1226	1.43	0.10	miR-554	1.09	0.20	miR-450b-5p	0.60	0.17
miR-384	1.42	0.23	miR-552	1.09	0.19	miR-587	0.60	0.08
miR-409-5p	1.41	0.42	miR-622	1.09	0.18	miR-1255a	0.59	0.19
miR-421	1.41	0.51	miR-556-3p	1.09	0.25	miR-1249	0.59	0.15
miR-1278	1.40	0.45	miR-557	1.08	0.42	miR-375	0.59	0.14
miR-423-3p	1.40	0.41	miR-559	1.08	0.34	miR-335	0.59	0.15
miR-551a	1.40	0.52	miR-1233	1.07	0.51	miR-1275	0.58	0.12
miR-424	1.39	0.38	miR-544	1.06	0.20	miR-365	0.57	0.13
miR-429	1.38	0.40	miR-564	1.06	0.27	miR-626	0.57	0.04
miR-330-3p	1.38	0.37	miR-567	1.06	0.37	miR-1296	0.56	0.09
miR-296-5p	1.38	0.13	miR-571	1.06	0.33	miR-1207-5p	0.54	0.15
miR-1184	1.38	0.50	miR-573	1.06	0.43	miR-1264	0.54	0.15
miR-448	1.37	0.41	miR-574-5p	1.05	0.51	miR-132	0.54	0.15
miR-449b	1.37	0.39	miR-577	1.05	0.20	miR-423-5p	0.53	0.24
miR-548j	1.34	0.59	miR-579	1.05	0.24	miR-607	0.53	0.17
miR-450b-3p	1.34	0.50	miR-616	1.05	0.02	miR-320d	0.53	0.21
miR-613	1.33	0.57	miR-135b	1.04	0.48	miR-520c-3p	0.52	0.16
miR-105	1.32	0.60	miR-338-5p	1.04	0.22			
miR-1224-5p	1.32	0.30	miR-125a-3p	1.04	0.03			
miR-1261	1.32	0.45	miR-522	1.03	0.04			
miR-453	1.31	0.50	miR-584	1.03	0.23			
miR-371-3p	1.31	0.65	miR-582-5p	1.03	0.12			
miR-455-3p	1.30	0.51	miR-581	1.03	0.10			
miR-483-3p	1.30	0.31	miR-21	1.03	0.50			
miR-484	1.30	0.40	miR-586	1.03	0.21			
miR-1245	1.28	0.02	miR-1205	1.02	0.19			
miR-486-5p	1.28	0.37	miR-588	1.02	0.16			
miR-485-5p	1.28	0.31	miR-590-3p	1.02	0.21			
miR-487b	1.28	0.41	miR-591	1.01	0.42			
miR-489	1.28	0.51	miR-383	1.01	0.02			
miR-490-5p	1.28	0.29	miR-593	1.01	0,3			
miR-491-5p	1.27	0.41	miR-596	1.00	0.41			
miR-493	1.27	0.50	miR-604	1.00	0.30			
miR-497	1.27	0.27	miR-602	1.00	0.42			
miR-548a-3p	1.26	0.45	miR-598	1.00	0,28			
miR-500	1.26	0.50	miR-1286	0.98	0.32			
miR-501-5p	1.26	0.42	miR-1285	0.98	0.38			
miR-502-5p	1.25	0.39	miR-1267	0.98	0.15			

Supplemental Table S1. MicroRNAs showing less than twofold differences in abundance in senescent relative to early-passage cells. RNA extracted from Y (pdl 22-25) and S (pdl 50-55) WI-38 cells was used to measure the levels of the microRNAs listed, using RT-qPCR (Materials and Methods). microRNA abundance was normalized to U1 snRNA levels. Data are the means and S.D. from three independent experiments.
