# Supplementary material for: MicroRNA profiling in human diploid fibroblasts uncovers miR-519 role in replicative senescence
Source: Aging (Albany NY). 2010 Jun 19;2(6):333–43. doi: 10.18632/aging.100159 (PMC2919253; doi:10.18632/aging.100159)
Supplement: Supplementary Table 2 [file aging-02-333-s002.pdf]

miR name	miRBase Acc No.	Primer Sequence	miR name	miRBase Acc No.	Primer Sequence
miR-96	MIMAT0000095	TTTGGCACTAGCACATTTTTGCT	miR-206	MIMAT0000462	TGGAATGTAAGGAAGTGTGTGG
miR-944	MIMAT0004987	AAATTATTGTACATCGGATGAG	miR-203	MIMAT0000264	GTGAAATGTTTAGGACCACTAG
miR-9	MIMAT0000441	UCUUUGGUUAUCUAGCUGUAUGA	miR-194	MIMAT0000460	TGTAACAGCAACTCCATGTGGA
miR-890	MIMAT0004912	TACTTGGAAAGGCATCAGTTG	miR-18b	MIMAT0001412	TAAGGTGCATCTAGTGCAGTTAG
miR-888	MIMAT0004916	TACTCAAAAAGCTGTCAGTCA	miR-18a	MIMAT0000072	TAAGGTGCATCTAGTGCAGATAG
miR-876-5p	MIMAT0004924	TGGATTTCTTTGTGAATCACCA	miR-1826	MIMAT0006766	ATTGATCATCGACACTTCGAACGCAAT
miR-874	MIMAT0004911	CTGCCCTGGCCCGAGGGACCGA	miR-15a	MIMAT0000068	TAGCAGCACATAATGGTTTGTG
miR-760	MIMAT0004957	CGGCTCTGGGTCTGTGGGGA	miR-155	MIMAT0000646	TTAATGCTAATCGTGATAGGGGT
miR-758	MIMAT0003879	UUUGUGACCUGGUCCACUAACC	miR-1537	MIMAT0007399	AAAACCGTCTAGTTACAGTTGT
miR-7	MIMAT0000252	TGGAAGACTAGTGATTTTGTTGT	miR-146b-3p	MIMAT0004766	TGCCCTGTGGACTCAGTTCTGG
miR-664	MIMAT0005949	UAUUCAUUUAUCCCCAGCCUACA	miR-141	MIMAT0000432	TAACACTGTCTGGTAAAGATGG
miR-663	MIMAT0003326	AGGCGGGGCGCCGCGGGACCGC	miR-140-5p	MIMAT0000431	CAGTGGTTTTACCCTATGGTAG
miR-658	MIMAT0003336	GGCGGAGGGAAGTAGGTCCGTTGGT	miR-140-3p	MIMAT0004597	TACCACAGGGTAGAACCACGG
miR-653	MIMAT0003328	GTGTTGAAACAATCTCTACTG	miR-135b	MIMAT0000758	TATGGCTTTTCATTCCTATGTGA
miR-651	MIMAT0003321	TTTAGGATAAGCTTGACTTTTG	miR-135a	MIMAT0000428	TATGGCTTTTTATTCCTATGTGA
miR-649	MIMAT0003319	AAACCTGTGTTGTTCAAGAGTC	miR-133b	MIMAT0000770	TTTGGTCCCCTTCAACCAGCTA
miR-641	MIMAT0003311	AAAGACATAGGATAGAGTCACCTC	miR-1323	MIMAT0005795	TCAAAACTGAGGGGCATTTTCT
miR-640	MIMAT0003310	ATGATCCAGGAACCTGCCTCT	miR-132	MIMAT0000426	TAACAGTCTACAGCCATGGTCG
miR-633	MIMAT0003303	CTAATAGTATCTACCACAATAAA	miR-1305	MIMAT0005893	TTTTCAACTCTAATGGGAGAGA
miR-628-5p	MIMAT0004809	ATGCTGACATATTTACTAGAGG	miR-1304	MIMAT0005892	TTTGAGGCTACAGTGAGATGTG
miR-626	MIMAT0003295	AGCUGUCUGAAAAUGUCUU	miR-1303	MIMAT0005891	TTTAGAGACGGGGTCTTGCTCT
miR-625	MIMAT0003294	AGGGGGAAAGTTCTATAGTCC	miR-1296	MIMAT0005794	TTAGGGCCCTGGCTCCATCTCC
miR-622	MIMAT0003291	ACAGTCTGCTGAGGTTGGAGC	miR-129-5p	MIMAT0000242	CTTTTTGCGGTCTGGGCTTGC
miR-618	MIMAT0003287	AAACTCTACTTGTCCTTCTGAGT	miR-129-3p	MIMAT0004605	AAGCCCTTACCCCAAAAAGCAT
miR-616	MIMAT0004805	AGTCATTGGAGGGTTTGAGCAG	miR-1291	MIMAT0005881	TGGCCCTGACTGAAGACCAGCAGT
miR-613	MIMAT0003281	AGGAATGTTCCTTCTTTGCC	miR-1289	MIMAT0005879	TGGAGTCCAGGAATCTGCATTTT
miR-607	MIMAT0003275	GTTCAAATCCAGATCTATAAC	miR-1288	MIMAT0005942	TGGACTGCCCTGATCTGGAGA
miR-605	MIMAT0003273	TAAATCCCATGGTGCCTTCTCCT	miR-1287	MIMAT0005878	TGCTGGATCAGTGGTTCGAGTC
miR-600	MIMAT0003268	ACTTACAGACAAGAGCCTTGCTC	miR-1286	MIMAT0005877	TGCAGGACCAAGATGAGCCCT
miR-587	MIMAT0003253	UUUCCAUAGGUGAUGAGUCAC	miR-1285	MIMAT0005876	TCTGGGCAACAAAGTGAGACCT
miR-584	MIMAT0003249	TTATGGTTTGCCTGGGACTGAG	miR-1284	MIMAT0005941	TCTATACAGACCCTGGCTTTTC
miR-577	MIMAT0003242	TAGATAAAATATTGGTACCTG	miR-1283	MIMAT0005799	TCTACAAAGGAAAGCGCTTTCT
miR-576-3p	MIMAT0004796	AAGATGTGGAAAAATTGGAATC	miR-1282	MIMAT0005940	TCGTTTGCCTTTTTCTGCTT
miR-572	MIMAT0003237	GTCCGCTCGGCGGTGGCCCA	miR-1281	MIMAT0005939	UCGCCUCCUCCUCUCCC
miR-569	MIMAT0003234	AGTTAATGAATCCTGGAAAGT	miR-1280	MIMAT0005946	TCCCACCGCTGCCACCC
miR-562	MIMAT0003226	AAAGTAGCTGTACCATTTGC	miR-128	MIMAT0000424	TCACAGTGAACCGGTCTCTTT
miR-561	MIMAT0003225	CAAAGTTTAAGATCCTTGAAGT	miR-1279	MIMAT0005937	TCATATTGCTTCTTTCT
miR-553	MIMAT0003216	AAAACGGTGAGATTTTGTTTT	miR-1278	MIMAT0005936	TAGTACTGTGCATATCATCTAT
miR-551a	MIMAT0003214	GCGACCCACTCTTGGTTTCCA	miR-1276	MIMAT0005930	TAAAGAGCCCTGTGGAGACA
miR-550	MIMAT0004800	AGTGCCTGAGGGAGTAAGAGCCC	miR-127-5p	MIMAT0004604	CTGAAGCTCAGAGGGCTCTGAT
miR-548p	MIMAT0005934	TAGCAAAAACTGCAGTTACTTT	miR-1275	MIMAT0005929	GUGGGGGAGAGGCUGUC
miR-548l	MIMAT0005935	AAAAGTAATTGCGGATTTTGCC	miR-1274a	MIMAT0005927	GTCCCTGTTCAGGCGCCA
miR-548k	MIMAT0005882	AAAAGTACTTGCGGATTTTGCT	miR-127-3p	MIMAT0000446	TCGGATCCGTCTGAGCTTGGCT
miR-548j	MIMAT0005875	AAAAGTAATTGCGGTCTTTGGT	miR-1273	MIMAT0005926	GGGCGACAAAGCAAGACTCTTTCTT
miR-548i	MIMAT0005935	AAAAGUAAUUGCGGAUUUUGCC	miR-1272	MIMAT0005925	GATGATGATGGCAGCAAATTCTGAAA
miR-548h	MIMAT0005928	AAAAGUAAUCGCGGUUUUUGUC	miR-1271	MIMAT0005796	CTTGGCACCTAGCAAGCACTCA
miR-548e	MIMAT0005874	AAAAACTGAGACTACTTTTGCA	miR-1270	MIMAT0005924	CTGGAGATATGGAAGAGCTGTGT
miR-548d-5p	MIMAT0005799	TCTACAAAGGAAAGCGCTTTCT	miR-1269	MIMAT0005923	CTGGACTGAGCCGTGCTACTGG
miR-548d-3p	MIMAT0003323	CAAAAACCACAGTTTCTTTTGC	miR-1268	MIMAT0005922	CGGGCGUGGUGGUGGGGG
miR-548c-5p	MIMAT0004806	AAAAGTAATTGCGGTTTTTGCC	miR-1267	MIMAT0005921	CCTGTTGAAGTGTAATCCCCA
miR-548c-3p	MIMAT0003285	CAAAAAUCUCAAUUACUUUUGC	miR-1266	MIMAT0005920	CCTCAGGGCTGTAGAACAGGGCT
miR-548b-3p	MIMAT0003254	CAAGAACCTCAGTTGCTTTTGT	miR-1265	MIMAT0005918	CAGGATGTGGTCAAGTGTTGTT
miR-548a-5p	MIMAT0004803	AAAAGUAAUUGCGAGUUUUACC	miR-1264	MIMAT0005791	CAAGTCTTATTTGAGCACCTGTT
miR-548a-3p	MIMAT0003251	CAAAACTGGCAATTACTTTTGC	miR-1263	MIMAT0005915	ATGGTACCCTGGCATACTGAGT
miR-545	MIMAT0003165	TCAGCAAACATTTATTGTGTGC	miR-1262	MIMAT0005914	ATGGGTGAATTTGTAGAAGGAT
miR-544	MIMAT0003164	ATTCTGCATTTTTAGCAAGTTC	miR-1261	MIMAT0005913	ATGGATAAGGCTTTGGCTT
miR-543	MIMAT0004954	AAACATTCGCGGTGCACTTCTT	miR-1260	MIMAT0005911	ATCCCACCTCTGCCACCA
miR-522	MIMAT0002868	AAAATGGTTCCCTTTAGAGTGT	miR-125b	MIMAT0000423	TCCCTGAGACCCTAACTTGTGA
miR-520e	MIMAT0002825	AAAGTGCTTCCTTTTTGAGGG	miR-125a-5p	MIMAT0000443	TCCCTGAGACCCTTTAACCTGTGA
miR-520d-3p	MIMAT0002856	AAAGUGCUUCUCUUUGGUGGGU	miR-125a-3p	MIMAT0004602	ACAGGTGAGGTTCTTGGGAGCC
miR-520c-3p	MIMAT0002846	AAAGTGCTTCCTTTTAGAGGGT	miR-1259	MIMAT0005910	ATATATGATGACTTAGCTTTT
miR-520b	MIMAT0002843	AAAGTGCTTCCTTTTAGAGGG	miR-1258	MIMAT0005909	AGTTAGGATTAGGTCGTGGAA
miR-520a-3p	MIMAT0002834	AAAGTGCTTCCCTTTGGACTGT	miR-1257	MIMAT0005908	AGTGAATGATGGGTTCTGACC
miR-519e	MIMAT0002829	AAGTGCCTCCTTTTAGAGTGTT	miR-1256	MIMAT0005907	AGGCATTGACTTCTCACTAGCT
miR-519c-3p	MIMAT0002832	AAAGTGCATCTTTTTAGAGGAT	miR-1255b	MIMAT0005945	CGGATGAGCAAAGAAAGTGGTT
miR-519b-3p	MIMAT0002837	AAAGTGCATCCTTTTAGAGGTT	miR-1255a	MIMAT0005906	AGGAUGAGCAAAGAAAGUAGAUU
miR-519a	MIMAT0002869	AAAGTGCATCCTTTTAGAGTGT	miR-1254	MIMAT0005905	AGCCTGGAAGCTGGAGCCTGCAGT
miR-518e	MIMAT0002861	AAAGCGCTTCCCTTCAGAGTG	miR-1253	MIMAT0005904	AGAGAAGAAGATCAGCCTGCA
miR-518d-3p	MIMAT0002864	CAAAGCGCTTCCCTTTGGAGC	miR-1250	MIMAT0005902	ACGGTGCTGGATGTGGCCTTT
miR-518c	MIMAT0002848	CAAAGCGCTTCTCTTTAGAGTGT	miR-1249	MIMAT0005901	ACGCCCUUCCCCCCCUUCUUCA
miR-518b	MIMAT0002844	CAAAGCGCTCCCCTTTAGAGGT	miR-1248	MIMAT0005900	ACCTTCTTGTATAAGCACTGTGCTAAA
miR-518a-3p	MIMAT0002863	GAAAGCGCTTCCCTTTGCTGGA	miR-1247	MIMAT0005899	ACCCGTCCCGTTCGTCCCCGGA
miR-517b	MIMAT0002857	TCGTGCATCCCTTTAGAGTGTT	miR-1246	MIMAT0005898	AATGGATTTTTGGAGCAGG
miR-516b	MIMAT0002859	ATCTGGAGGTAAGAAGCACTTT	miR-1245	MIMAT0005897	AAGTGATCTAAAGGCCTACAT

Supplemental Table S2. Primers used for RT-qPCR detection of the microRNAs in Figures 2 and 3 and in supplemental Figure S1.
